# Development of comorbidities in type 2 diabetes between 2005 and 2017 using German claims data

**DOI:** 10.1038/s41598-021-90611-x

**Published:** 2021-05-27

**Authors:** Batoul Safieddine, Stefanie Sperlich, Jelena Epping, Karin Lange, Siegfried Geyer

**Affiliations:** 1 Medical Sociology Unit OE 5420, Hannover Medical School, Carl-Neuberg-Str. 1, 30625 Hannover, Germany; 2 Medical Psychology Unit OE 5430, Hannover Medical School, Carl-Neuberg-Str. 1, 30625 Hannover, Germany

**Keywords:** Diseases, Health care

## Abstract

Against the background of increasing life expectancy over time, several hypotheses have been proposed on the way morbidity has been developing. In type 2 diabetes (T2D), previous research suggests that morbidity compression could be ruled out due to increasing prevalence and life expectancy with T2D over time. Understanding how the health state in individuals with T2D is developing would help identify whether morbidity expansion or a dynamic equilibrium pattern applies for this disease. This study aims to answer the following questions: (1) How do the number and the prevalence of T2D concordant comorbidities develop over time? (2) What does this imply in terms of morbidity development in T2D in Germany? The study used claims data from a statutory health insurance provider in Lower Saxony, Germany. Period prevalence of T2D concordant comorbidities was examined for the periods 2005–2007, 2010–2012 and 2015–2017 in 240,241, 295,868 and 308,134 individuals with T2D respectively. The effect of time period on the number and prevalence of comorbidities was examined by means of (ordered) logistic regression. The age-adjusted predicted probabilities of more severe cardiovascular diseases (CVDs) decreased over the three periods while those of less severe CVDs and other vascular diseases increased significantly in men and women and among all examined age-groups. Predicted probability of having at least one more comorbidity over time also increased significantly among all examined groups. While less and more severe CVDs exhibited different developmental patterns, the results of the study point towards morbidity expansion in T2D. Future studies should focus on mechanisms that contribute to these trends.

## Introduction

Beside the evidence of increasing life expectancy in many countries around the world^1,2^, the health state and morbidity during the gained life time have been an area of investigation in the public health sector. Since the late 1970s, three alternative hypotheses have been proposed on how morbidity could be developing over time. In 1977, Ernest Gruenberg presented the hypothesis of morbidity expansion. His hypothesis suggests that while medical progress is associated with an improvement in life expectancy, it does not necessarily imply compression of morbidity due to a more prolonged time spent with morbidity and chronic disease^3^. A few years later, James Fries formulated a more optimistic approach designating compression in morbidity. His hypothesis suggests that due to better primary prevention and living conditions, onset of morbidity is postponed towards higher age groups and that at a faster pace than the increase of life expectancy. According to Fries, the impact of primary prevention on morbidity exceeded that on mortality thus leading to morbidity compression^4,5^. In 1982, Kenneth Manton reposed an intermediate position by formulating the hypothesis of dynamic equilibrium. It suggests that while advances in modern medicine are associated with an increase in the rates of chronic diseases and disabilities, they also are associated with better quality of life due to improved coping, independency and social inclusion^6^.

Yet, the above mentioned hypotheses were not formulated for specific diseases but rather focused on general morbidity. While various studies examined the development of morbidity drafting different development patterns, many of them did not focus on specific diseases as outcomes^7,8^. It therefore remains open how the development of morbidity will look like when specific diseases are considered. Type 2 Diabetes (T2D) is a disease of global concern due to its relatively high prevalence rate and the associated public health burden^9,10^. In Germany, research examining the development of morbidity in the context of T2D has been recently initiated. Two studies were conducted using the same large dataset of claims data from Lower Saxony *(the same data base used in this study)*. The first one investigated the change in T2D age at onset and age at death between the years 2005–2013. It showed that in the younger age group (19–39 years), age at onset was declining over time while age at death was increasing for the age cohort 60–79 years^11^. The second study investigated the change in life expectancy with T2D from 2005 to 2014. The study found that life expectancy with T2D increased steadily and significantly for both genders and all age groups considered. The study also found that prevalence rates increased during the examined period with a 3.8% increase in the age standardized prevalence in both men and women^12^. The results of the preceding studies suggest that in this population, compression of morbidity is ruled out for the case of T2D. Nevertheless, the results do not necessarily signpost an expansion of morbidity. Based on Manton’s hypothesis^6^, if individuals with T2D were adjusting their lifestyle to the disease and thus have less complications and better quality of life over time, a dynamic equilibrium would instead mirror the development of morbidity. Therefore, the question remains unanswered whether the hypothesis of morbidity expansion really applies to individuals with T2D.

The quality of life in individuals with T2D is primarily affected by associated complications and comorbidities^13^. If not well managed, T2D can cause severe cardio-vascular and neurological complications^14^ that, beside the distress of daily coping and disease management, can significantly deteriorate the quality of life in individuals with T2D^15–17^. Not only do T2D complications affect the quality of life of individuals, but also do they have a public health impact due to the associated financial burden. It is estimated that in Germany, one in every ten euros of health care expenditure is currently spent on direct medical costs of T2D. These costs amount to 16.1 billion euros annually with two-thirds attributed to the treatment of diabetes secondary diseases^18^. Against this background, this study investigates how comorbidities are developing over time in T2D. This would help to better identify the morbidity development pattern in this very common disease, which is in turn essential for future planning of public health relevant issues such as budget planning and the postponement of retirement age.

Following the disease management program for T2D that was introduced in 2003 in Germany, it can be expected that since then, individuals with T2D are better coping with the disease due to the implementation of evidence-based guidelines and education measures^19,20^. Therefore, we hypothesize that the prevalence of diabetes comorbidities would be decreasing over time, which would support the hypothesis of dynamic equilibrium in the context of T2D. If on the contrary the prevalence of T2D comorbidities either remained stable or increased over time, this would support the hypothesis of morbidity expansion. However, while to our knowledge only a few studies examined the trends in T2D comorbidities, their results provide contradicting evidence on their developmental patterns. A recent study from the UK examined the trend of a large list of T2D comorbidities and reported increasing prevalence of depression but no change in the prevalence of T2D concordant conditions from the year 2007 through 2017^21^. Another study from Sweden reported a substantially declining incidence of cardiovascular diseases among individuals with diabetes from the year 1998 through 2014^22^. A US study however found contradicting results where it pointed towards an increasing trend in the prevalence of CVD comorbidities but not in other comorbidities^23^.

In this study, using claims data from a large statutory health insurance provider in the state of Lower Saxony-Germany, we aim to investigate the development of comorbidities in T2D over time during the period 2005–2017. Due to the scope of the study, we chose to focus on T2D concordant comorbidities. Specifically, we aim to answer the following questions:

1. How does the number of T2D concordant comorbidities in individuals with T2D develop over time?
2. How does the prevalence of T2D concordant comorbidities develop over time?
3. What do the answers of the two above questions indicate in terms of morbidity development in T2D in Germany?

## Methods

### Data

This study is based on claims data from a large statutory health insurance provider in Lower Saxony, Germany: the Allgemeine Ortskrankenkasse Niedersachsen (AOKN). In Germany, health insurance is mandatory and 90% of the population has health insurance under the regulation of statutory health insurances with insurance premiums that depend on personal income^24^. Statutory health insurance operates under public law, and health care plans are defined according to nationwide regulations. AOKN insures around one third of the population in the state of Lower Saxony with a somewhat varying socioeconomic distribution from the German population^25^. The data by AOKN is mainly collected for accounting purposes and are currently available for the years 2005 to 2017. The data includes demographic information, in- and outpatient diagnoses, medical prescriptions and medical treatments.

The access to the data was granted by a signed contract with the AOKN under consideration and adherence of data security and privacy issues. Basic plausibility and data management were performed by the AOKN. Pseudonymised datasets were then made available at the institute of the research team.

### Definition of T2D cases and comorbidities

This study was performed using data from insured women and men with T2D aged 18 years and older. All diagnoses are coded according to the German-modified 10th version of the International Classification of Diseases (ICD-10-GM), which slightly differs from the international version.

Diabetes mellitus with its various types is assigned the ICD-10-GM codes E10-E14. While T2D is assigned the code E11, erroneous or double coding (different diabetes codes for the same patient) could be detected in the data. Thus, in order to improve the accuracy of defining T2D cases, certain criteria were applied: First, individuals who had E11 as the most frequently coded diagnosis during the observation period were considered to be having T2D. When the most frequent diagnosis was E14 which refers to the category “undefined”, individuals were also considered to have T2D because T2D constitutes almost 90% of all diabetes cases^9^. In addition, since type-1 diabetes always involves insulin intake, individuals with E10 most frequently coded but without any insulin prescription were also considered T2D cases. Moreover, individuals insured for more than one quarter but have a diabetes diagnoses in only one quarter were not considered as eligible cases. For the identification of T2D diagnoses, both confirmed outpatient diagnoses and primary and secondary inpatient diagnoses were considered. Outpatient diagnoses with the data fields “suspected” or “ruled out” were not considered.

The list of T2D-concordant comorbidities was defined according to previous research^14,26^. Comorbidities included were: hypertension, hyperlipidemia, cardiac insufficiency, angina pectoris, myocardial infarction, stroke, retinopathy, nephropathy and polyneuropathy which were also defined according to the corresponding ICD-10-GM codes. Obesity was not considered in the study because it is only very rarely coded by physicians. Where it was not clear which codes correspond to the comorbidities in question, the definition of codes was done according to that of the Scientific Institute of AOKN (WIdO)^27^. WIdO publishes consecutive reports on different diseases and the utilization of medical services using claims data of the AOKN^28^. Similar to the above-mentioned definition criterion for T2D, cases of chronic comorbidities such as hypertension were considered eligible if they had diagnoses in at least two quarters, given that they are insured for more than one quarter. Confirmed outpatient and/or primary and secondary inpatient diagnoses were considered for the identification of comorbidities depending on the type of comorbidity. The list of ICD-10-GM codes as well as the types of diagnoses used to define the examined comorbidities is found in Supplementary Table S1.

### Time periods

The development of comorbidities in T2D was examined over three time periods with equal intervals and gaps as follows: Period one involved the years 2005–2007, period two involved the years 2010–2012 and period three the years 2015–2017. In order to limit bias, T2D as well as all included comorbidities were newly defined using the same defining criteria described above in each of the three periods. This would improve the comparability of the three periods because it allows for the same potential errors in the identification of T2D and comorbidities in all three periods. Including three 3-year periods to examine the development of comorbidities was convenient in order to allow for subgroup analyses (three age groups and gender) since some examined comorbidities such as stroke or myocardial infarction could be rare for certain subgroups and the number of events within only one year would not be sufficient for analysis. Considering 3-year time periods was also a dynamic solution since first, having longer periods would lead to a greater overestimation of the period prevalence when considering the observation time in the denominator (weighting the denominator according to person time to adjust for censoring), and second, having shorter periods (for example 2-year periods) would create more subgroups (five time periods, nine comorbidities, three age groups and two gender groups) which would affect the clarity and presentation of the results. Since the data is available for 13 years at the time of the study, it was chosen to have three observation periods with equal intervals and equal gaps in between in order to improve their comparability while still considering the earliest and the latest years available to examine the temporal development of T2D comorbidities. Therefore, the years 2008–2009 and 2013–2014 were not considered in the analysis*.*

### Statistical analyses

All statistical analyses in this study were stratified by gender and three age groups: 18–45 years, 46–64 years and 65 + years in order to provide a more comprehensive picture on the development of comorbidities in T2D among men and women over different age groups. In the first line of analysis, the change in the number of comorbidities in individuals with T2D was examined. The number of comorbidities was grouped into the four categories: “0”, “1”, “2” and “3 + ” (3 or more). Since the outcome is ordinal, ordered logistic regression was applied while adjusting for within cluster variation in order to correct for the possible existence of autocorrelation. In this analysis, the outcome was the number of comorbidities with the four above mentioned categories and the main independent variable was “time period” while adjusting for age within each age group and insurance duration. Ordered logistic regression provides an Odds Ratio (OR) indicating the odds of being one category higher in the outcome (in our case 0 to 1, 1 to 2 or 2 to 3 + comorbidities) for the examined group (in our case the periods 2010–2012 and 2015–2017) compared to the control group (2005–2007).

In the second line of analysis, the single comorbidities were considered. First, period prevalence was calculated for each of the nine examined T2D comorbidities. Since not all individuals are insured (and thus observed) for the same amount of time in the examined periods, denominators were weighted according to person-time. Second, in order to examine whether the change in the prevalence of comorbidities was significant over the three periods, logistic regression analysis was applied. In this analysis, it was also adjusted for the within cluster variation due to possible effects of having individuals in more than one period which can be associated with autocorrelation. Comorbidities were grouped into three categories in order to provide a lucid overview. The first category comprised less severe cardiovascular comorbidities (CVD-Less Severe) summarizing hypertension, hyperlipidemia and cardiac insufficiency. The second category included more severe cardiovascular comorbidities (CVD-More Severe): myocardial infarction, stroke and angina pectoris. The third category (Other Vascular Diseases) included the other three examined comorbidities: retinopathy, nephropathy and polyneuropathy. The models included the grouped comorbidities variables as outcome, the time period as the main independent variable and adjusted for age within each age category and duration of observation while applying margins at means to ease the interpretation. In this analysis, Prevalence Ratios (PRs) were used instead of ORs to interpret the effect of time on comorbidities. This is because ORs tend to either overestimate (when OR > 1) or underestimate (when OR < 1) effects when dealing with outcomes of more than 10% prevalence rate^29^.

In all regression analyses mentioned above, the results were also displayed graphically using predicted probabilities. Predicted probabilities were estimated using margins at means adjusting for age and insurance duration. They have the benefit of revealing adjusted effects that can be interpreted with higher accuracy compared to prevalence rates^30^.

All analyses were performed with the software: STATA v15.1. All methods were performed in accordance with the relevant guidelines and regulations.

### Ethics approval and consent to participate

No ethics approval is required for this study. The analyses were performed using a pseudonymized pre-existing claims dataset. Their scientific use is regulated by German law in the German Civil Code “Bürgerliches Gesetzbuch”. The data protection officer of the Local Statutory Health Insurance of Lower Saxony-AOK Niedersachsen (Germany) has given permission to use the data for scientific purposes.

## Results

Our study population consists of individuals with T2D in the three observed periods: 2005–2007, 2010–2012 and 2015–2017. The study was done on 240241, 295868 and 308134 individuals with T2D in periods one, two and three respectively. Basic population characteristics in the three examined periods are presented in Table 1.

**Table 1:** Population characteristics.

	2005–2007	2010–2012	2015–2017
N	240,241	295,868	308,134
**Age**			
18–45 years n (%)	10,893 (4.5)	13,745 (4.7)	14,724 (4.8)
46–64 years n (%)	58,923 (24.5)	80,621 (27.3)	86,585 (28.1)
65 + years n (%)	170,425 (70.9)	201,502 (68.1)	206,825 (67.1)
**Gender**			
Women n (%)	135,816 (56.5)	159,544 (53.9)	161,222 (52.3)
Men n (%)	104,425 (43.5)	136,324 (46.1)	146,912 (47.7)
**Insurance duration**			
Mean (SD)	1009 (231)	1005 (227)	1003 (242)

In the three observed periods, the period prevalence of T2D increased considerably in both genders and all age groups. Since examining the prevalence trend of T2D was not a major part of this study, results of this ad hoc analysis are illustrated in Supplementary Table S2 and Supplementary Figure S3.

### Prevalence of single comorbidities

Table 2 presents the period prevalence rates of the nine included comorbidities stratified by age group, gender and time period. The results show that comorbidities with the highest prevalence rates in all examined groups and all three periods were hypertension and hyperlipidemia, while myocardial infarction and stroke were the least prevalent. Besides that, the prevalence of the three comorbidities: myocardial infarction, angina pectoris and stroke either decreased or only very slightly increased over the three periods. On the other hand, the period prevalence rates of all other examined comorbidities increased over the three time periods. The highest increase was observed in nephropathy and polyneuropathy which almost doubled between the periods: 2005–2007 and 2015–2017 for most of the examined groups (Table 2).

**Table 2:** Period prevalence of single comorbidities in T2D patients (in percent) by age group, sex and time period.

Age	Comorbidity	Males			Females		
		2005–2007	2010–2012	2015–2017	2005–2007	2010–2012	2015–2017
18–45 years	Myocardial infarction	1.10	1.02	1.55	0.45	0.35	0.29
	Angina pectoris	2.25	2.00	1.61	1.26	1.22	0.59
	Stroke	0.74	0.59	0.66	0.59	0.43	0.49
	Hypertension	40.58	48.90	55.35	36.03	41.15	45.87
	Hyperlipidemia	30.61	32.67	36.15	20.51	21.54	23.05
	Cardiac insufficiency	2.21	2.63	3.04	0.93	1.27	2.14
	Retinopathy	2.19	2.00	2.82	2.05	1.59	2.29
	Nephropathy	3.16	4.32	5.79	2.60	3.32	4.33
	Polyneuropathy	7.23	8.81	9.98	7.17	9.92	10.60
46–64 years	Myocardial infarction	2.76	2.88	3.04	1.18	1.07	1.17
	Angina pectoris	5.80	5.56	4.66	3.87	3.22	2.64
	Stroke	2.13	2.06	2.15	1.31	1.30	1.20
	Hypertension	66.44	74.41	81.10	70.27	76.86	81.95
	Hyperlipidemia	43.79	50.20	55.89	37.77	43.52	48.36
	Cardiac insufficiency	7.40	9.22	11.78	6.21	6.86	8.06
	Retinopathy	4.08	4.46	5.55	4.19	4.29	5.36
	Nephropathy	6.62	9.63	13.09	4.92	7.39	10.93
	Polyneuropathy	12.61	18.28	22.02	13.55	18.49	22.80
65 + years	Myocardial infarction	4.54	4.70	4.47	3.21	3.08	2.70
	Angina pectoris	8.93	8.83	7.24	6.73	6.45	5.18
	Stroke	5.34	5.06	5.04	5.13	4.73	4.46
	Hypertension	84.91	95.71	100	90.79	98.91	100
	Hyperlipidemia	46.57	58.35	66.61	44.75	55.51	62.60
	Cardiac insufficiency	25.88	29.17	34.80	33.89	33.50	36.56
	Retinopathy	6.02	7.35	9.71	6.21	7.34	9.21
	Nephropathy	19.36	27.67	38.22	15.86	24.09	36.03
	Polyneuropathy	15.49	23.51	32.74	15.99	23.59	32.55

### Development of the number of comorbidities over time

**Figure 1:**
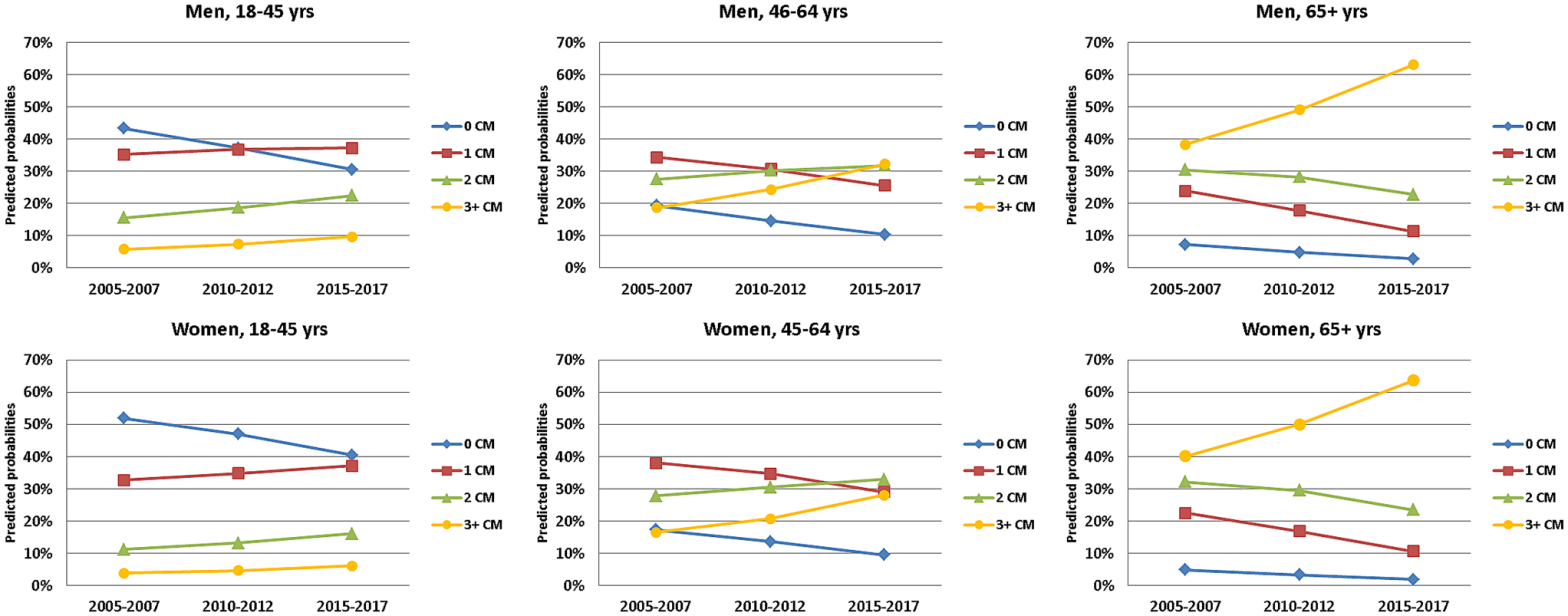
Predicted probabilities of the number of comorbidities in T2D over time period, stratified by age and gender. *CM* comorbidity.

Holding age and insurance duration at their mean values within each examined group, the predicted probability of having no comorbidities decreased over the three periods in both genders and all three examined age groups among our population of individuals with T2D (Fig. 1). However, the development of the three other categories (1, 2 and 3 + comorbidities) was not identical among the three examined age groups. In men and women aged 18–45 years, the probability of having at least one comorbidity increased by 2 to 6%. In the age group 46–64 years, the probability of having only one comorbidity decreased by 8 to 9% in men and women respectively, while the probabilities of having 2 or 3 + comorbidities increased. In individuals aged 65 + years, the probabilities of have only 1 or only 2 comorbidities decreased, while the probability of having 3 + comorbidities increased considerably in men and women, from around 38% in 2005–2007 to 64% in 2015–2017 (Fig. 1).

The ordered logistic regression analysis showed that in all three age groups and among both genders, there was a significant increase in the number of comorbidities over time. In this analysis, the outcome variable was the number of comorbidities which was illustrated by the four categories: 0, 1, 2 and 3 + comorbidities. In individuals with T2D aged 18–45 years, being in the second period was associated with 29% and 22% more risk of having at least one additional comorbidity compared to the period 2005–2007 for men and women respectively. The risk of having more comorbidities increased to 74% and 59% for the period 2015–2017 compared to 2005–2007 for men and women respectively. In the age group 46–64 years, the increase in the risk of having more comorbidities over time was more prominent. While men and women in this group respectively had 40 and 32% higher odds of having at least one more comorbidity in the second time period, the risk almost doubled in the time period 2015–2017. The increase in the risk for having a higher number of comorbidities was most pronounced in the age group 65 + years. In this age group, men and women were almost 1.5 times more likely to have at least one additional comorbidity in 2010–2012, and had up to 2.75 times higher risk in 2015–2017 (Table 3).

**Table 3:** Odds ratios and confidence intervals of the effect of time period on the number of comorbidities in T2D patients, stratified by age and gender, estimated by means of ordered logistic regression adjusting for within cluster variation.

		18–45 years n (men) = 19,340 n (women) = 20,022		46–64 years n (men) = 127,461 n (women) = 98,668		65 + years n (men) = 240,860 n (women) = 337,892	
		OR	CI	OR	CI	OR	CI
Men	2005–2007	1	–	1	–	1	–
	2010–2012	1.29	1.22—1.38	1.40	1.37—1.43	1.55	1.52—1.58
	2015–2017	1.74	1.63—1.86	2.07	2.01—2.12	2.75	2.70—2.80
Women	2005–2007	1	–	1	–	1	–
	2010–2012	1.22	1.14—1.30	1.32	1.29—1.36	1.49	1.47—1.51
	2015–2017	1.59	1.49—1.69	1.97	1.91—2.03	2.62	2.57—2.66

Adjusted for age (within each age group) and insurance duration. Outcome: number of comorbidities in four categories: 0, 1, 2 and 3 + .

All odds ratios were significant at p < 0.001.

### Development of the three comorbidity groups

The development of the two CVD comorbidity groups: less severe and more severe exhibited different patterns. The risk of having at least one of the less severe comorbidities (hypertension, hyperlipidemia or cardiac insufficiency) increased significantly over the three periods in both men and women and all three age groups (Table 4). The increase in the risk for this comorbidity group was most pronounced in individuals aged 18–45 years with 12–15% higher risk in 2010–2012 and 26–27% higher risk in 2015–2017 for women and men respectively. On the other hand, the risk of having more severe comorbidities (myocardial infarction, stroke or angina pectoris) did not show a significant change for a few of the examined groups: 18–45 year old men over the three time periods, 18–45 year old women over the second time period and 46–64 year old men over the second time period. For all other examined groups, the risk of having more severe CVD decreased significantly. The decreased risk was more pronounced in women than in men with up to 21% decreased risk in the period 2015–2017. The risk of having other vascular diseases (retinopathy, nephropathy and polyneuropathy) also increased significantly over the three time periods for men and women of all age groups. The increase in the risk was even more pronounced than that observed for less severe CVD, but was quite similar in both genders. In 18–45 year olds, there was up to 49% more risk in 2015–2017 compared to 2005–2007. The increase in the risk was even higher in the two older age groups with up to 76% increased risk for 65 + year old women in the 2015–2017 (Table 4).

**Table 4:** Prevalence ratios and confidence intervals of the effect of time period on the comorbidity-index variables (1) severe CVD, (2) less severe CVD and (3) other vascular diseases, stratified by age and gender as estimated by means of logistic regression adjusting for within cluster variation. Adjusted for age (within each age group) and insurance duration.

			18–45 years n (men) = 19,340 n (women) = 20,022			46–64 years n (men) = 127,461 n (women) = 98,668			65 + years n (men) = 240,860 n (women) = 337,892		
			PR	CI	p	PR	CI	p	PR	CI	p
CVD—less severe	Men	2005–2007	1	–		1	–		1	–	
		2010–2012	1.15	1.11—1.18	< 0.001	1.08	1.07—1.09	< 0.001	1.06	1.05—1.06	< 0.001
		2015–2017	1.27	1.23—1.31	< 0.001	1.15	1.14—1.16	< 0.001	1.09	1.09—1.09	< 0.001
	Women	2005–2007	1	–		1	–		1	–	
		2010–2012	1.12	1.08—1.16	< 0.001	1.06	1.06—1.07	< 0.001	1.04	1.04—1.05	< 0.001
		2015–2017	1.26	1.21—1.31	< 0.001	1.11	1.11—1.12	< 0.001	1.06	1.06—1.06	< 0.001
CVD—more severe	Men	2005–2007	1	–		1	–		1	–	
		2010–2012	0.93	0.75—1.11	0.483	0.98	0.94—1.02	0.295	0.95	0.93—0.97	< 0.001
		2015–2017	1.02	0.82—1.22	0.840	0.92	0.88—0.96	< 0.001	0.84—0.88	< 0.001	
	Women	2005–2007	1	–		1	–		1	–	
		2010–2012	0.89	0.67—1.12	0.369	0.89	0.83—0.95	< 0.001	0.93	0.91—0.95	< 0.001
		2015–2017	0.64	0.46—0.82	< 0.05	0.79	0.74—0.85	< 0.001	0.81	0.79—0.83	< 0.001
Other vascular diseases	Men	2005–2007	1	–		1	–		1	–	
		2010–2012	1.23	1.11—1.35	< 0.001	1.33	1.30—1.36	< 0.001	1.30	1.28—1.31	< 0.001
		2015–2017	1.48	1.34—1.63	< 0.001	1.62	1.58—1.66	< 0.001	1.65	1.63—1.67	< 0.001
	Women	2005–2007	1	–		1	–		1	–	
		2010–2012	1.30	1.17—1.43	< 0.001	1.27	1.24—1.31	< 0.001	1.34	1.33—1.36	< 0.001
		2015–2017	1.49	1.34—1.63	< 0.001	1.59	1.54—1.63	< 0.001	1.76	1.74—1.78	< 0.001

CVD-less severe: hypertension, cardiac insufficiency or hyperlipidemia.

CVD-more severe: stroke, myocardial infarction or angina pectoris.

Other vascular diseases: retinopathy, nephropathy or polyneuropathy.

**Figure 2:**
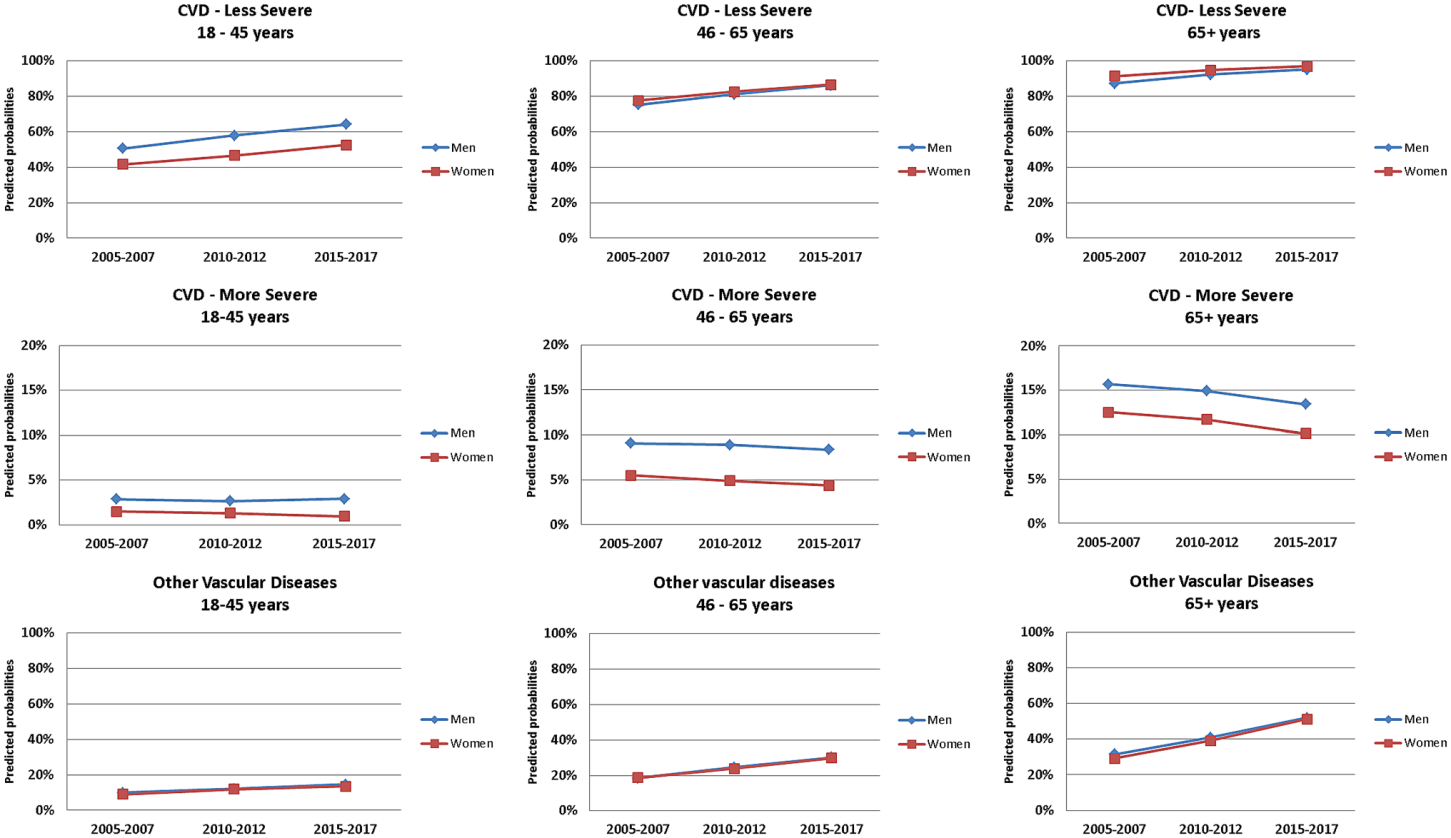
Predicted probabilities of having less severe CVD, more severe CVD and other vascular diseases in T2D over time period, stratified by age and gender.

These results could also be observed through the predicted probabilities illustrated in Fig. 2. While the probability of having less severe CVD was already relatively high in the first time period, an increasing trend in the adjusted predicted probabilities is noticeable. Keeping age and insurance duration at their mean values within each examined age group and gender, the probability of having less severe CVD was as high as 42–50% for 18–45 year olds, 75–78% for 46–65 year old and around 90% for 65 + year olds in 2005–2007. These probabilities increased by over 10% for all examined groups in the time period 2015–2017 reaching up to 95–97% for 65 + year old men and women respectively. On the other hand, the adjusted probabilities of having more severe CVD remained more or less constant for 18–45 year olds but decreased by a few percent points for the two older age groups. The probability of having at least one comorbidity from the comorbidity group “other vascular diseases” showed an increasing trend for all age groups, and the probabilities were rather similar among men and women. The increase in the predicted probability for this comorbidity group was up to 5% for 18–45 year olds, 11% for 46–65 year olds and 32% for 65 + year olds.

## Discussion

In an attempt to understand the morbidity development pattern in the context of T2D, this study investigated the development of T2D concordant comorbidities in a population of statutory insured individuals in Lower Saxony, Germany. Since the disease management program was introduced in 2003 in Germany, it was hypothesized that comorbidities would be decreasing due to better secondary prevention^20^, which would support the dynamic equilibrium hypothesis of Manton^6^. However, this study showed that the number of T2D concordant comorbidities as well as the predicted probabilities of less severe CVDs and other vascular diseases are considerably increasing over time in men and women with T2D between the years 2005 and 2017.

The results of previous studies examining the trends in comorbidities in T2D are not fully consistent. While Zhang et al. (2010) showed a rising trend only in CVDs among other investigated T2D comorbidities in the US, Rawshani et al. (2017) reported a substantially declining incidence of CVDs in T2D in Sweden. In addition, Nowakowska et al. (2019) reported only an increase in depression but no change in other T2D concordant comorbidities over time in the UK^21^. One possible explanation for the contradicting results could be related to the undiagnosed T2D cases, the so-called dark figure. Since T2D often remains undetected until comorbidities have been developed, the prevalence of comorbidities could be overestimated. Nevertheless, there is evidence that the prevalence of undiagnosed T2D cases has been decreasing in Germany with a 1,8% reduction between the years 1997 (3.8%) and 2011 (2%)^31^. This implies that the denominator is increasing when estimating the prevalence of comorbidities leading to less overestimation of the prevalence of comorbidities in Germany.

Another explanation for contradicting results of the temporal development of comorbidities could be the issue of “up-coding” associated with claims data. It has been hypothesized that more diagnoses are being coded from the side of the physicians especially after implementation of the disease management program in 2011 which provides adequate follow up and screening for comorbidities in T2D patients. Yet, this cannot be confirmed as to our knowledge, no scientific evidence exists on up-coding so far. Furthermore, the results of other survey-based studies in Germany are in favor of the results obtained in this study. A recent survey based study by Sperlich et al. investigated the change in disability rates in T2D patients in Germany and found that, among all age groups, disability rates have been significantly increasing. The study showed that the increase in disability rates was significantly attributed to the increase in depression, obesity and other comorbidities^17^. This supports the results of our study which point towards an expansion of morbidity in T2D rather than a dynamic equilibrium. *Even though the prevalence of more severe CVDs did not significantly increase, but rather decreased over time in most examined subgroups, the described results of this study still support expansion of morbidity*. This is because more severe CVDs are often followed by severe disabilities, shorter lifespan or mortality^32^. On the other hand, life expectancy with diabetes has been rising significantly^12^ and age at onset of T2D has been shown in previous studies to be decreasing^11^. These findings, together with the finding of this study about the increasing trend in the number and the prevalence of most T2D concordant comorbidities indicate that T2D patients are in fact living longer with more disease related complications over time.

Yet, it is questionable why the prevalence of more severe comorbidities decreased while other comorbidities increased significantly. In Germany, previous studies point towards morbidity compression in severe outcomes such as myocardial infarction^33,34^ and stroke^35,36^. In addition, a recent study done on disability trends in Europe showed that overall disability increased by 2.37% between 2006 and 2016, while there was only a slight increase (0.44%) in severe disabilities^37^. The robust increase found in this study in the prevalence of less severe CVDs which act as risk factors for health outcomes such as myocardial infarction and stroke can be reasoned by several arguments. It can be speculated that a higher prevalence of medical drugs intake, accompanied by deterioration in lifestyle risk factors such as nutrition, smoking and lack of physical activity could be the reason behind such a development. The more common intake of drugs could postpone or prevent serious health events, but not necessarily lead to morbidity compression due to prolonging the time spent with disability. A German survey based longitudinal study examined the development of cardio-metabolic risk factors and found that the prevalence of the use of antidiabetic, cholesterol lowering and antihypertensive medications rose significantly between 1990 and 2011^38^. At the same time, the study reported a rising prevalence of smoking and obesity which are major risk factors for diabetes and its comorbidities. Nevertheless, more recent analyses would be essential to validate these results and draw adequate conclusions on the mechanisms leading to the described developments of T2D comorbidities. Further studies are planned to investigate the trend of the use of medications among individuals with T2D in our study population. Investigating the trends in lifestyle risk factors in our study population is also essential but would only be possible through matching survey data and applying multi-level analysis due to the lack of lifestyle information in claims data. In addition, this study did not stratify by or adjust for socioeconomic status in order to observe the actual development apart from the temporal change in socioeconomic factors that could play a role. Since T2D^39^ as well as the risk factors for comorbidities^40^ are largely affected by social factors, understanding social inequalities in the development of comorbidities would provide a more comprehensive picture of the underlying mechanisms.

### Strengths and limitations

This study was done using claims data that includes a large population of statutory insured individuals in the state of Lower Saxony, thus providing adequate power. All diagnoses are captured with no effects of recall or selection bias that are usually associated with survey-based studies. However, due to the scope of the study, only T2D concordant comorbidities were considered. Other comorbidities such as depression are also common and can affect the quality of life and morbidity situation of individuals with T2D. Thus, considering the development of T2D discordant comorbidities is necessary to confirm the conclusions of this study. Second, since T2D is often diagnosed after individuals have already developed related complications, the prevalence of comorbidities could have been overestimated. In addition, since AOKN have a somewhat varying socioeconomic distribution from the general population in Germany^25^, the results cannot be fully generalized and a socioeconomically stratified analysis should follow.

## Conclusions

This study found that the age adjusted predicted probabilities of more severe CVDs decreased while those of less severe CVDs and other vascular diseases increased over time in men and women with T2D. The results point towards morbidity expansion in the population of type 2 diabetics in the state of Lower Saxony, Germany. Future studies should focus on mechanisms that contribute to these trends. Examining the temporal developments of the use of medications, T2D risk factors as well as socioeconomic status are the following steps in understanding the underlying mechanisms.

## Supplementary Information

Supplementary Information.

## Data Availability

The data underlying this study belong to the Allgemeine Ortskrankenkasse Niedersachsen (AOKN-General Local Health Insurance of Lower Saxony). The data are not publically available due to protection of data privacy of the insured individuals by the AOKN. Interested researchers can send data access requests to Dr. Jona Stahmeyer at the AOKN using the following e-mail address: Jona.Stahmeyer@aok.nds.de. The authors did not have any special access privileges.

## Abbreviations

AOKN: Allgemeine Ortskrankenkasse Niedersachsen
CVD: Cardiovascular disease
ICD-10-GM: International Classification of Disease, version 10, German modification
OR: Odds ratio
PR: Prevalence ratio
T2D: Type 2 diabetes
WidO: Scientific Institute of AOK (Wissenschaftliches Institute der AOK)
