# Supplementary material for: Development of comorbidities in type 2 diabetes between 2005 and 2017 using German claims data
Source: Sci Rep. 2021 May 27;11:11149. doi: 10.1038/s41598-021-90611-x (PMC8159920; doi:10.1038/s41598-021-90611-x)

**Development of Comorbidities in Type 2 Diabetes between 2005 and 2017 using German Claims Data**

**Supplementary Tables and Figures**

**Supplementary Table S1.** ICD-10-GM codes of included comorbidities.

| **Comorbidity** | **ICD-10-GM Codes** | **Type of Diagnosis** |
| --- | --- | --- |
| **Myocardial Infarct** | I21 | Primary inpatient |
| **Stroke** | I60-I64 | Primary inpatient |
| **Angina Pectoris** |  |  |
| *Stable Angina* | I20.1, I20.8, I20.9 | Primary and secondary inpatient & confirmed outpatient |
| *Unstable Angina* | I20.0 | Primary and secondary inpatient & confirmed outpatient |
| **Hypertension** | I10-I15 | Primary and secondary inpatient & confirmed outpatient |
| **Hyperlipidemia** | E78 | Primary and secondary inpatient & confirmed outpatient |
| **Cardiac Insufficiency** | I50 | Primary and secondary inpatient & confirmed outpatient |
| **Retinopathy*** | H36 | Confirmed outpatient |
| **Nephropathy** | N18, N19, N08.3 | Primary and secondary inpatient & confirmed outpatient |
| **Polyneuropathy**** | G63 | Confirmed outpatient & secondary inpatient |
|  | Epilepsy medications: N03AX16, N03AX12 or N03AF01 but with no epilepsy diagnosis: G40-G41  Depression medications: N06AA09, N06AX21 but with no depression diagnosis: F30-F39 |  |

*No inpatient diagnoses were present in the data

**No primary inpatient diagnoses were present in the data

**Supplementary Table S2.** Prevalence of Type 2 Diabetes in the time periods 2005-2007, 2010-2012 and 2015-2017. Stratified by gender and five age groups.

| **Men** | | | | | | |
| --- | --- | --- | --- | --- | --- | --- |
|  |  | **18-30 years** | **31-45 years** | **46-60 years** | **61-80 years** | **81+ years** |
|  | T2D | 436 | 5205 | 23923 | 62576 | 12285 |
|  | Total* | 152242 | 218750 | 219325 | 222583 | 36423 |
| ***Prevalence*** | **2005-2007** | 0.29% | 2.38% | 10.91% | 28.11% | 33.73% |
|  | T2D | 625 | 6129 | 31695 | 78140 | 19735 |
|  | Total* | 188951 | 218769 | 266277 | 239558 | 49351 |
| ***Prevalence*** | **2010-2012** | 0.33% | 2.80% | 11.90% | 32.62% | 39.99% |
|  | T2D | 811 | 6134 | 35136 | 78350 | 26481 |
|  | Total* | 212632 | 232248 | 290462 | 238740 | 59537 |
| ***Prevalence*** | **2015-2017** | 0.38% | 2.64% | 12.10% | 32.82% | 44.48% |
| **Women** | | | | | | |
|  |  | **18-30 years** | **31-45 years** | **46-60 years** | **61-80 years** | **81+ years** |
|  | T2D | 727 | 4525 | 18503 | 71941 | 40120 |
|  | Total* | 151884 | 209504 | 216271 | 296213 | 115507 |
| ***Prevalence*** | **2005-2007** | 0.48% | 2.16% | 8.56% | 24.29% | 34.73% |
|  | T2D | 1169 | 5822 | 24079 | 81284 | 47190 |
|  | Total* | 178786 | 205620 | 253016 | 293027 | 124836 |
| ***Prevalence*** | **2010-2012** | 0.65% | 2.83% | 9.52% | 27.74% | 37.80% |
|  | T2D | 1489 | 6290 | 25969 | 75580 | 51894 |
|  | Total* | 195666 | 212512 | 275075 | 277505 | 130364 |
| ***Prevalence*** | **2015-2017** | 0.76% | 2.96% | 9.44% | 27.24% | 39.81% |

*Rounded according to person-time.

**Supplementary Figure S3.** Prevalence of Type 2 Diabetes in the time periods 2005-2007, 2010-2012 and 2015-2017. Stratified by gender and five age groups.
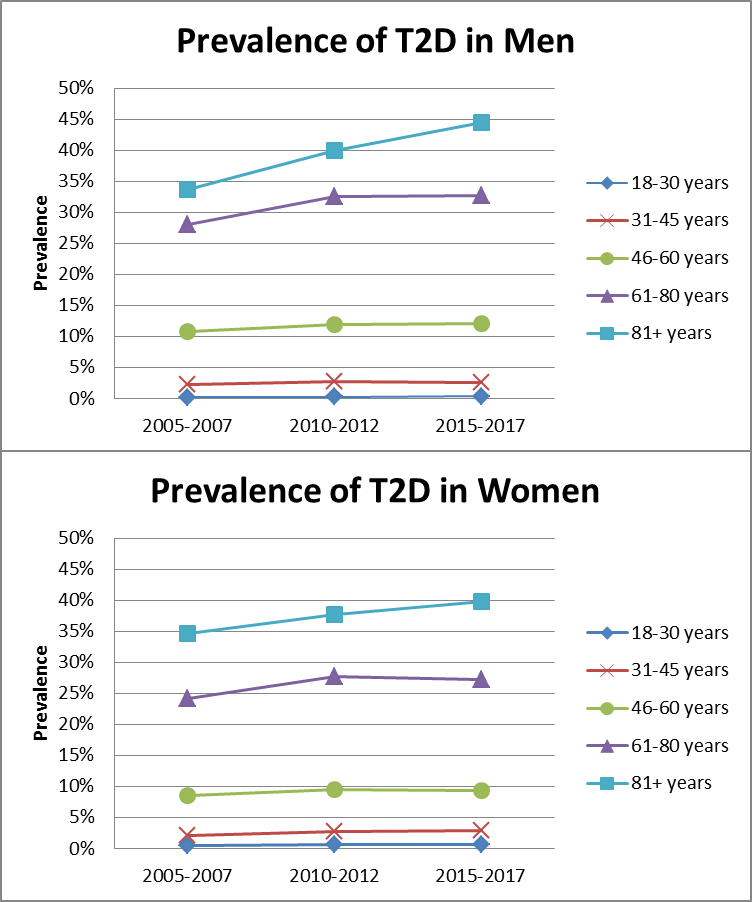

Supplement: Supplementary file 1 — Supplementary Information. [file 41598_2021_90611_MOESM1_ESM.docx]
